# Acute and long-term psychosocial consequences in grandparents when a grandchild is diagnosed with cancer – the GROKids Project: a population-based mixed-methods study protocol

**DOI:** 10.1186/s40359-023-01309-w

**Published:** 2023-09-18

**Authors:** Gisela Michel, Peter Francis Raguindin, Cristina Priboi, Anica Ilic, Pauline Holmer, Katrin Scheinemann, the GROKids Project, Nicolas von der Weid, Pierluigi Brazzola, Jochen Roessler, Marc Ansari, Manuel Diezi, Maja Beck-Popovic, Freimut Schilling, Jeanette Greiner, Heinz Hengartner

**Affiliations:** 1 Faculty of Health Sciences and Medicine, University of Lucerne, Alpenquai 4, 6005 Lucerne, Switzerland; 2 Division of Hematology/Oncology, Children’s Hospital of Eastern Switzerland, St Gallen, Switzerland; 3 Department of Pediatrics, McMaster Children’s Hospital and McMaster University, Hamilton, Canada; 4 Oncology/Hematology, University Children’s Hospital Basel, Basel, Switzerland; 5 Emato-Oncologia pediatrica, Istituto Pediatrico della Svizzera Italiana, Ospedale Regionale di Bellinzona e Valli, Bellinzona, Switzerland; 6 Paeditaric Oncology/Haematology, Children’s University Hospital, Inselspital Bern, Bern, Switzerland; 7 Pediatric Onco-hematology Unit, University Hospital Geneva, Geneva, Switzerland; 8 Pediatric Onco-hematology Unit, Centre Hospitalier Universitaire Vaudois, Lausanne, Switzerland; 9 Paeditaric Haematology and Oncology, Lucerne Cantonal Hospital, Lucerne, Switzerland; 10 Pädiatrische Onkologie-Hämatologie, Kinderspsital, Kantonsspital Aarau, Aarau, Switzerland

**Keywords:** Grandparent, Psychological outcomes, Elderly, Aging, Childhood cancer

## Abstract

**Background:**

Grandparents play a crucial role in providing their families with love, support, and wisdom, often also supporting them in practical and financial ways. The psychosocial effects experienced by grandparents when a grandchild is diagnosed with an illness can be significant, including increased stress, anxiety, grief, and disruptions in their own lives. Yet, the experience of grandparents is often overlooked in the literature.

**Methods/design:**

The GROKids Project aims to investigate how grandparents are affected by a grandchild's cancer diagnosis. It employs a mixed-methods approach and consists of three studies: a longitudinal cohort study (Study 1) and a qualitative study (Study 2) involving grandparents of children with a recent cancer diagnosis, and a cross-sectional study (Study 3) of grandparents of childhood cancer survivors. Study 1 covers four time points over two years after the cancer diagnosis, while Study 2 explores the lived experiences of a subsample of these grandparents. Study 3 collects data from grandparents of childhood cancer survivors diagnosed 3 to 10 years ago. Participants are recruited across eight pediatric oncology centers in Switzerland, and through patient advocacy and support groups. Eligibility criteria include having a grandchild diagnosed with cancer and being fluent in German, French, or Italian.

Study procedures involve requesting grandparents’ contacts from eligible families, and later contacting grandparents, providing study information, obtaining informed consent, and sending out questionnaires by post or online. Reminder calls and mails are used to improve response rates. Data analysis includes multilevel regression (Study 1), thematic analysis (Study 2), and regression analyses (Study 3). Various validated questionnaires are used to assess physical health and overall well-being, psychological health, internal, and external factors.

**Discussion:**

This project addresses the gaps in understanding the psychosocial effects on grandparents having a grandchild diagnosed with cancer. It utilizes a comprehensive approach, including multiple methodologies and considering the broader family context. The project’s strengths lie in its mixed-methods design, longitudinal approach, and inclusion of the perspectives of the sick children, siblings, and parents, besides grandparents. By gaining a more profound understanding of grandparents' experiences, researchers and healthcare professionals can develop targeted interventions and support services to address grandparents’ unique needs.

**Supplementary Information:**

The online version contains supplementary material available at 10.1186/s40359-023-01309-w.

## Background

Grandparents play a crucial role in the family structure, providing their grandchildren with love, support, and wisdom [1]. Many grandparents support their families in practical, emotional, and financial ways. Care for grandchildren may range from occasional care to legal guardianship. For many families, childcare by grandparents is essential to allow parents to attend paid work [2, 3]. In Switzerland, almost one-third of grandmothers care for their grandchildren at least once a week, when they are under six years old; later, this proportion decreases to about 15% [4]. A European study showed that grandparents looked after their grandchildren for 57 h per month on average [5]. In Switzerland, the annual number of hours of grandparental care for grandchildren was estimated to 160 million hours, and its monetary equivalent to CHF 8.2 million in 2016 [4]. In cases of difficulty, grandparents may take on additional grandparental role duties for their grandchildren, including providing economic support for the family and helping to look after young grandchildren.

When a child is acutely ill, grandparents play an essential role for the affected families and can care for siblings at home or the sick child in the hospital [6]. Therefore, grandparents need the appropriate resources, such as good physical and emotional health and finances [7], to support their children’s families, especially with the potentially increased burden of an ill grandchild. The literature on the psychosocial effects of childhood illness on grandparents reveals a range of emotional and psychological experiences [8]. When a grandchild is diagnosed with an illness, grandparents often experience heightened stress, anxiety, and grief [9, 10]. They may struggle with helplessness, guilt, and concern for their grandchild's well-being. Furthermore, they may face significant disruptions in their own daily lives, including financial strain [11], alterations in social activities [9, 12], and adjustments in caregiving roles [13]. However, most studies were exploratory, using qualitative analysis to identify the impact on grandparents. Few studies used surveys, and none have done longitudinal analyses or utilized a holistic approach through mixed methods.

There is a huge knowledge gap on the effects on health and wellbeing on the aging population for when their grandchild suffers a life-threatening disease. Understanding the specific contributions and challenges grandparents face in these situations is important for providing effective support systems and health promotion. Our studies will shed light on the often-neglected experiences of grandparents, highlighting their unique needs and challenges. By gaining a deeper understanding of their emotional and psychological well-being, researchers and healthcare professionals can develop targeted interventions and support services to address these needs. Recognizing and addressing the psychosocial effects on grandparents may benefit their mental health and contribute to a more comprehensive and holistic approach to supporting families facing a childhood illness.

## Methods/design

**Fig. 1:**
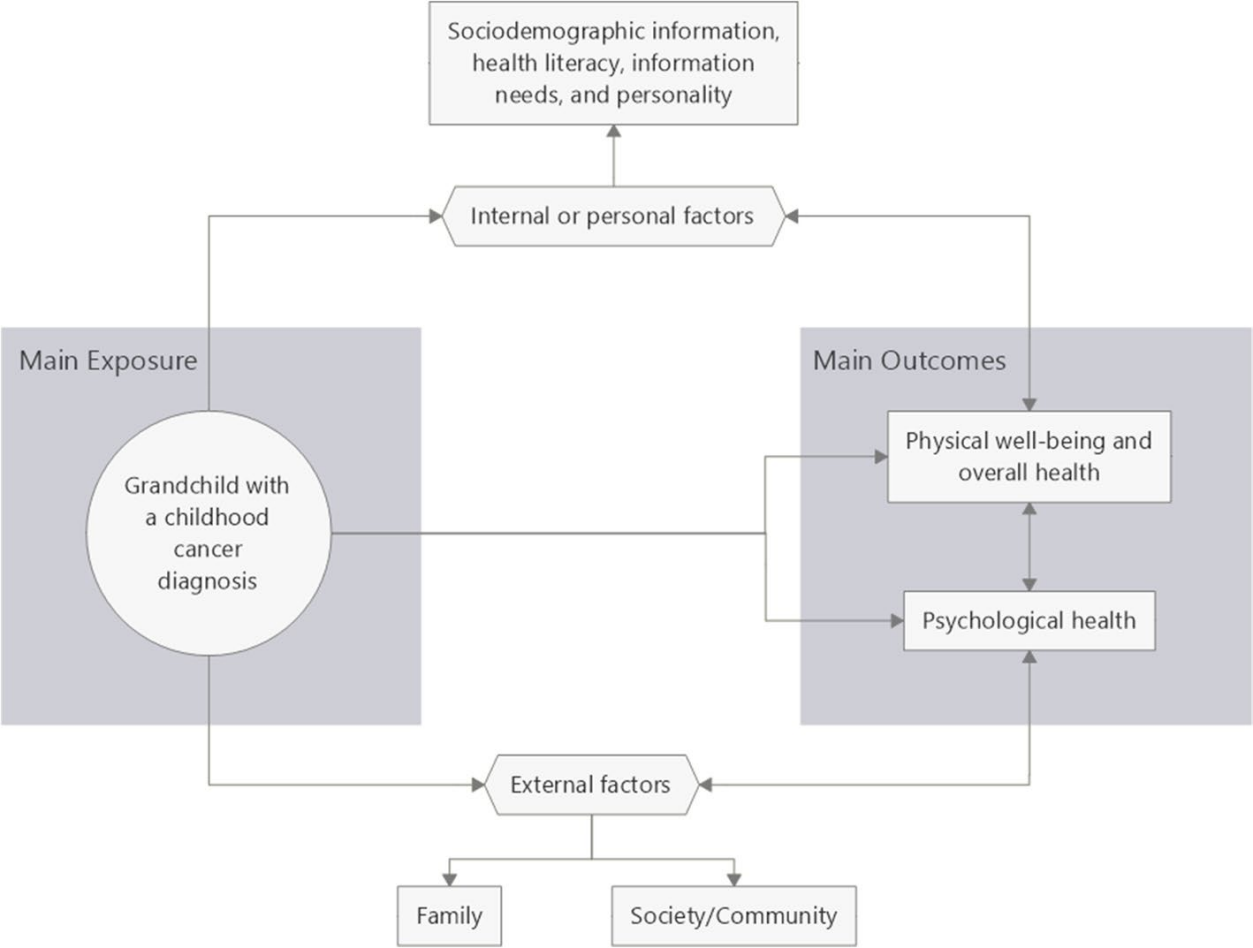
Conceptual framework. This diagram shows the population, the main outcomes observed, and the confounding factors in the association (see Table 3 for details of the tools/questionnaires used for measurement)

The overall objective is to investigate how grandparents are affected by a childhood cancer diagnosis of a grandchild (Fig. 1). The study will address the following questions: (a) How and to what extent are grandparents involved in caring for their grandchildren during and shortly after cancer treatment? (b) What are the acute consequences for grandparents of a grandchild diagnosed with cancer? (c) What are the long-term consequences of childhood cancer on grandparents?

**Fig. 2:**
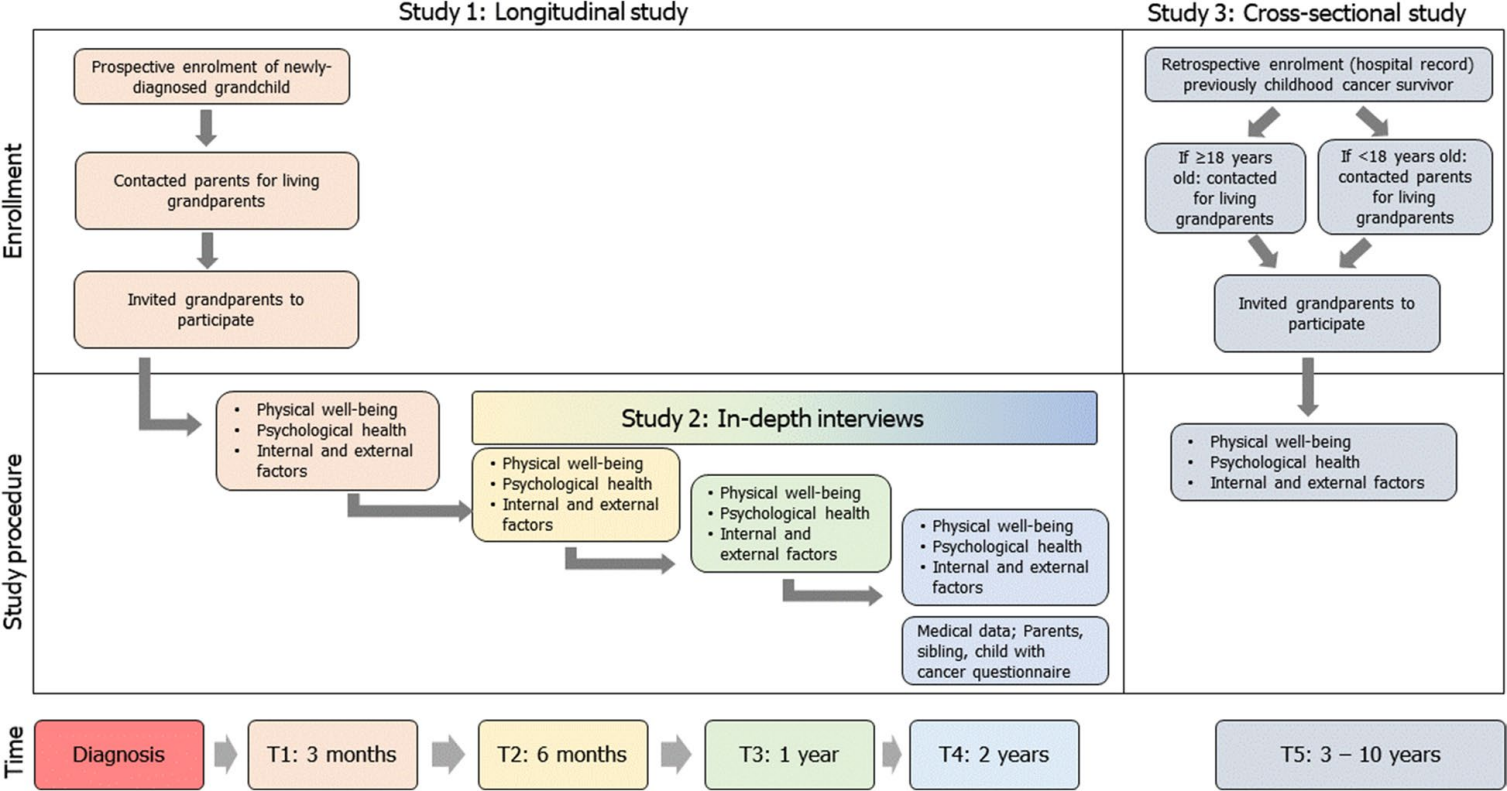
Schematic diagram on grandparent enrollment and follow-up (See Table 3 for details on tools/questionnaires used at each observation period)

This is an ongoing observational study using a mixed-methods approach combining: a longitudinal cohort study (Study 1), a qualitative semi-structured interview study (Study 2), and a cross-sectional survey study (Study 3) (Fig. 2). Study 1 repeatedly assesses participants at four time points, namely, at three months (T1), six months (T2), one year (T3), and two years (T4) after the grandchild’s cancer diagnosis. Follow-up length was chosen because after two years cancer therapy should be completed for all cancer types. Study 2 will explore the lived experience of grandparents during the grandchild’s cancer therapy. Study 3 will collect data from grandparents of childhood cancer survivors who are 3 to 10 years (T5) from the diagnosis (completed treatment; Table 1). This population-based study will enroll participants from eight pediatric oncology centers across Switzerland (Table 2). The study started enrollment in 25 November 2020 and the last data collection (follow-up) is until 19 December 2024.

**Table 1:** Comparison of the different components of the three studies of the GROKids project in detail

	Study 1	Study 2	Study 3
Time from the diagnosis of childhood cancer	0–2 years (Immediate and acute effects)	0–2 years (Immediate and acute effects)	3–10 years (Long-term effects)
Type of study	Quantitative	Qualitative	Quantitative
Method	Cohort study using self-administered questionnaires	Semi-structured interviews	Cross-sectional study using self-administered questionnaires
Identification of eligible families	• Prospective enrollment of new cases in clinics • Volunteer participation	Same as Study 1	• Through patient pediatric oncology patient list • Volunteer participation
Eligibility criteria	For grandchild with cancer • Diagnosis of any childhood cancer within the last three months, except “watch and wait” patients • Patient aged ≤ 18 years at diagnosis • Patient is a resident of Switzerland (or near the borders) at the start of the study • Patient is under active treatment • Treatment in Switzerland • At least one grandparent or step-grandparent is alive For grandparents • Fluent in German, French, or Italian	Same as Study 1	For grandchild cancer survivor • Diagnosis of any type of childhood cancer • Has been diagnosed 3 to 10 years ago and is off treatment, based on the date of diagnosis • Survivor aged ≤ 18 years at diagnosis • Survivor was a resident of Switzerland at the diagnosis • Survivor was under treatment (no watch-and-wait patients) • Treatment in Switzerland • At least one grandparent or step-grandparent is alive For grandparents • Fluent in German, French, or Italian
Time points	T1 – 3 months T2 – 6 months T3 – 1 year T4 – 2 years	Between T2-T4	T5-3 to 10 years from the diagnosis
Main Outcomes	• Acute and chronic disease • Pain • Quality-of-life • Depression and anxiety • Post-traumatic stress • Adaptations to stress • Partner and family relationships	• Experiences • Help and support • Health and well-being • Daily life and employment • Relationship • Advice to grandparents and positive outcomes	• Acute and chronic disease • Pain • Quality-of-life • Depression and anxiety • Post-traumatic stress • Adaptations to stress • Partner and family relationships
Analysis	Repeated measures using multilevel regression	Thematic content analysis	Univariable and multivariable regression

**Table 2:** Collaborating centers

Participating institution	City	Canton	Regional Language
Pädiatrische Onkologie-Hämatologie, Kinderspital, Kantonsspital Aarau (Aarau Cantonal Hospital)	Aarau	Aargau	German
Onkologie/Hämatologie, Universität-Kinderspital beider Basel (University-Children’s Hospital in Basel)	Basel	Basel Stadt	German
Emato-Oncologia pediatrica, Istituto Pediatrico della Svizzera Italiana, Ospedale Regionale di Bellinzona e Valli (Bellinzona Regional Hospital)	Bellinzona	Ticino	Italian
Pädiatrische Hämatologie/Onkologie, Universitätsklinik für Kinderheilkunde, Inselspital (Bern University Hospital)	Bern	Bern	German
Unité d’onco-hématologie pédiatrique, Hôpitaux universitaires de Genève (Geneva University Hospital)	Geneva	Geneva	French
Unité d’hématologie-oncologie pédiatrique, Service de pédiatrie, Département femme-mère-enfant, Centre hôpitalier universitaire vaudois (Lausanne University Hospital)	Lausanne	Vaud	French
Pädiatrische Hämatologie und Onkologie, Kinderspital, Luzerner Kantonsspital (Lucerne Cantonal Hospital)	Lucerne	Lucerne	German
Hämatologie/Onkologie, Ostschweizer Kinderspital (East Switzerland Children’s Hospital)	St. Gallen	St. Gallen	German

### Eligibility criteria

Studies 1 and 2 focus on the acute consequences of a childhood cancer diagnosis for grandparents. Eligible childhood cancer cases include: (a) newly diagnosed patients, age ≤ 18 years), (b) undergoing treatment for cancer, and (c) treatment in Switzerland (at one of the participating centers). Each participating center will provide study information to eligible parents or grandparents. Interested parents or grandparents provide their own and family / grandparents contact details that the study team uses for initial contact. From this, we will contact and select grandparents who can understand one of the official languages in the country (German, French, Italian).

Study 3 focuses on the long-term consequences of a childhood cancer diagnosis on grandparents. Each participating center will identify childhood cancer survivors in their hospital registry. We use the following eligibility criteria: (a) availability of contact information of parents (if the survivor is < 18 years old) or survivors (if survivors are at least 18 years old), (b) cancer diagnosis 3 to 10 years before recruitment, and (c) has undergone treatment and is alive at study. Each participating center sends out study information and an invitation to join the study. Parents of CCS under 18 years old or adult survivors share the contact information of the grandparents to the study team. Grandparents will be contacted directly by the study team, and included if they understand one of the official languages in the country (German, French, Italian).

To expand the enrollment, we will distribute advertisements to advocacy groups and parents support groups in Switzerland. Interested participants can contact the study team by phone, mail, or email. The eligibility of the volunteer participant will be reviewed for any of the three studies.

### Study procedures

With the first contact from the study team, grandparents receive an information letter including the study's aims, the team's contact details, and a consent form for study participation. After providing their consent, grandparents receive the respective questionnaire for the study they are included in. We also provide the contact information of a psychologist, should any participant need counseling. All information and questionnaires are sent by post (with an option for online follow-up questionnaires in Study 1), and are available in German, French, and Italian. For Study 1, questionnaires are sent at T1, T2, T3, and T4. At T4, separate questionnaires are also sent to the parents, the child diagnosed with cancer and siblings (if available). For Study 3, questionnaires are sent after consent is provided. We send reminder mails in case of no response for four weeks. These reminders are done three times before classifying as a dropout. The reasons for dropout will be recorded, if available. Several cohort retention strategies are used to prevent dropouts in Study 1, namely, (a) newsletters, (b) appreciation cards and small gifts, and (c) follow-up calls by study staff.

Participants have an option to complete all questionnaires online (Qualtrics XM, Provo, Utah). If participants complete the questionnaire on paper, the study staff enter the responses in the online questionnaire. Personal data or personally identifiable information are encoded separately and saved in a secured server. Data quality and audits are performed weekly.

For Study 2, we will invite grandparents eligible for Study 1 for an interview. It will be a semi-structured interview following the interview guide in Appendix Table S1. Interested participants will be asked when and where the interview will take place. Interviews will be done individually, unless they want to be interviewed as a couple. Audio recording will be done and will be transcribed verbatim. The interview will be conducted in German, French, or Italian.

### Measures

Information for studies 1 and 3 is collected through self-administered questionnaires (Table 3), which are based on a previously published theoretical framework on the grandparents-grandchildren relationship considering the family context [14]. Information on diagnosis and treatment of the grandchild is obtained from participating centers if the survivor or their parents provide consent.

**Table 3:** Domains investigated in the study (including the standardized questionnaires as applicable)^a^

Domain	Tool	Study 1				Study 3	Scales/information	References
		T1	T2	T3	T4	T5		
Physical and overall wellbeing	Health-Related Quality of Life (SF-36)	x				x	Self-report questionnaire on health status. Contains 36 items with 8 subscales, namely, vitality, physical functioning, bodily pain, general health perception, physical role functioning, emotional role functioning, social role functioning, and mental health	[15–17]
	European Quality of Life questionnaire (EQ-5D-5L)	x	x	x	x	x	Self-report questionnaire on health status validated for use in health-economic assessments. This measures 5 dimensions, namely mobility, self-care, usual activities, pain/discomfort, and anxiety/depression	[18]
	Chronic diseases – Perrig & Merlo	x			x	x	This consists of 11 most common chronic disorder answerable by “yes” or “no” by the respondents	[19]
Psychological health	Brief Symptom Inventory (BSI-18)	x	x	x	x	x	Scale consists of 18 items with 3 subscales, namely, somatization, depression, and anxiety	[20, 21]
	Worry and Anxiety Questionnaire (WAQ)	x	x	x	x	x	Scale with 15 items and 2 subscales, namely, cognitive criteria or somatic criteria	[22]
	Impact of Event Scale (IES-R)		x	x	x	x	Measures post-traumatic stress using 22 items comprising of 3 subscales, namely, intrusive symptoms, avoidance, and hyperarousal	[23]
	Perceived Stress Scale (PSS)	x	x	x	x	x	Contains 10 items that measure the level of stress. No subscales	[24]
	Post Traumatic Growth Inventory (PTGI)	x				x	Measures posttraumatic growth and self-improvement after stress, containing 21 items with 5 subscales, namely, personal strength, new possibilities, improved relationships, spiritual growth, and appreciation for life	[25]
	Connor-Davidson Resilience Scale (CD-RISC 10 & 25)	(10)	(10)	(10)	(10)	(25)	Contains 25 items (for cross-sectional) or 10 items (for longitudinal) which measures someone’s ability to recover from stress	[26, 27]
Internal/personal factors	Sociodemographic information	(45)	(28)	(28)	(28)	(27)	Demographic information, such as age, sex, education, employment, living arrangement, income. It also includes the support received by the grandparents	[28, 29]
	Information needs	x	x	x	x	x	Validated scale with 21 items that measure the information needs and preferences of grandparents regarding their grandchild’s diagnosis of cancer	[30]
	European Health Literacy Survey Questionnaire (HLS-EU-Q12)			x		x	A 12-item version from the original 47 item questionnaire HLS-EU-Q47) that examines healthcare, disease prevention, and health promotion	[31]
	Big Five Inventory of personality (BFI-K)			x		x	A German version of the Big Five Inventory using 21 items assessing five personalities extraversion, agreeableness, conscientiousness, neuroticism, and openness)	[32]
External factors	Multidimensional Scale of Perceived Social Support (MSPSS)	x		x			A self-report measure of social support with 12 items from three sources (subscale), namely, family, friends, and significant other/partner	[33]
	Family relationships (FaBel)		x				German version of the “Impact on Family” Scale. Measures the parent’s or caregiver’s perception of the impact of pediatric illness on the family. This consists of 18 items with subscales include financial impact, familial-social impact, personal strain, and mastery	[34, 35]
	Adult-specific relationship attachment scales for partner relationship	x	x	x	x	x	A 14-item questionnaires given to partners, analyzed in pairs, and has 2 subscales, namely, security of attachment and perceived available support	[36, 37]

^a^Study 1- Longitudinal study focusing on acute biopsychosocial effects; Study 3- Cross-sectional study focusing on long term effects; Study 1 timepoints include T1 at 3 months, T2 at 6 months, T3 at 1 year, and T4 at 2 years; Study 3 timepoint (T5) is at long-term defined as 3–10 years after diagnosis. Numbers in the parenthesis indicate the modified number of items in the standardized questionnaires

#### Physical health and overall well-being outcomes

Physical health refers to absence of symptoms, disability, impairment, or chronic conditions with adequate energy level for daily functioning [38]. Overall wellbeing refers to health in a biopsychosocial framework, including quality-of-life. These outcomes are measured using self-reported health conditions and physical, mental and overall health perception of the participants using the following instruments: Health-related Quality-of-Life (SF-36) [15, 16]; the European Quality of Life questionnaire (EQ-5D-5L) [39]; and chronic disease checklist [19].

#### Psychological health outcomes

Psychological health refers to psychological, mental and behavioral outcomes that are affected by the grandchild’s cancer diagnosis. We include validated self-administered questionnaires to measure and operationalize psychological health outcomes at different time points. These questionnaires include: Brief Symptom Inventory (BSI-18) to measure somatization, depression and anxiety and overall psychological distress; Worry and Anxiety Questionnaire (WAQ) to measure worries and anxiety [22]; Impact of Event Scale (IES-R) to measure post-traumatic stress [23]; Perceived Stress Scale (PSS) to quantify the level of stress [24]; and Post Traumatic Growth Inventory (PTGI) [25] and Connor-Davidson Resilience Scale (CD-RISC 10 & 25) [26] to measure adaptations to stress.

#### Internal/personal factors

There are multiple personal factors that may affect the physical, psychological, and overall well-being of grandparents. We also collect several of these internal factors that may act as confounders on the well-being of grandparents. These tools include: Swiss Federal Statistical Office census questions to determine sociodemographic profile [40]; Information needs questionnaire to measure the perceived needs [30]; European Health Literacy Survey Questionnaire (HLS-EU-Q12) to determine health literacy [31]; and Big Five Inventory of personality (BFIK) to assess personality [32].

#### External factors (family and society)

The family and society may affect the well-being of grandparents. As such, we also collect information on social constructs surrounding the participants. These include: Multidimensional Scale of Perceived Social Support (MSPSS) to assess the support from family, friends and partner [33]; Impact on Family Relationships (FaBel) to measure the impact of illness on family [34]; and Relationship Attachment Scales [36] to measure partner relationship.

#### Sociodemographic information

Other demographic and economic information are obtained through a subset of questionnaires adapted from the questionnaires of the Swiss Federal Statistical Office [40]. We developed questions to evaluate self-reported changes in income, employment, housing and other variables due to cancer. Sociodemographic characteristics are known to be determinants of health and well-being.

#### Other information

For Study 1, we also collect additional information from the child with cancer, siblings, and parents. This will provide a broader context of the family dynamics that the grandparents are within. These questionnaires include sociodemographic data, self-reported health and well-being, and psychological status (Table 4).

**Table 4:** Other information obtained from the family of the grandparents (taken at the end of follow-up, longitudinal study, Study 1)

	Information/Questionnaires^a^
Grandchild (patient)^b^	1. Kidscreen-27^c^
	2. Open questions on family relationship and contact with grandparents
Siblings^b^	1. Kidscreen-27^c^
	2. Open questions on family relationship and contact with grandparents
Parents	1. Health-Related Quality of Life (SF-36)
	2. Brief Symptom Inventory (BSI-18)
	3. Perceived Stress Scale (PSS)
	4. Impact of Event Scale (IES-R)
	5. Adult-specific relationship attachment scales for partner relationship
	6.Worry and Anxiety Questionnaire (WAQ)
	7. Sociodemographic information
	8. Open questions on family relationship and contact with grandparents

^a^Please refer to Table 3 for specific details on the tools used

^b^We only enrolled grandchild (patient) and siblings who are 10 years old and above

^c^Quality of life measures as self-reported by children and adolescents. Consisting of 27 items [41, 42]

### Data analysis

For Study 1, we plan to analyze using multilevel (hierarchical) regression analysis with individuals and families as a cluster (repeated measures approach). We will explore the changes across time, with and without time-varying covariates. Time interaction will be fitted to account for changes across time. Determinants of outcome changes will also be explored using multivariable regression and/or by fitting interaction terms (between exposure and risk factor).

For the qualitative study (Study 2), the analysis of interview transcripts will follow the principles of qualitative thematic analysis according to guidelines developed by Braun and Clarke [43]. The transcripts will be entered into the qualitative data analysis software ATLAS.ti. (Scientific Software Development GmbH, Berlin). After familiarizing with the data, initial codes will be generated to search for major themes systematically. Each transcript will be coded once and then enriched with new codes inductively evolving from the transcripts. Subsequently, the coded segments are systematized and categorized.

For Study 3, regression analyses will determine the risk factors for physical and psychological outcomes. Univariable and multivariable regression analyses will be done with covariates chosen according to the theoretical framework by Davey, et al., and Wakefield, et. al. on the grandparent-grandchild relationship [10, 14]. All calculations will be done using Stata 17.0 (StataCorp, Texas), using two-tailed tests, with *p*-values < 0.05 considered statistically significant. Adjustments for multiple testing will be done, if appropriate.

### Sample size

Power analyses for Health-related Quality-of-Life (HRQoL) and psychological distress showed that the sample size of 100 is sufficient to detect a difference of 3 points on the SF-36 (a small effect) measuring HRQoL [17] between grandparents and the general population with a power of > 0.80 (alpha 0.05; two-sided), and a difference of 10% in the prevalence of individuals with clinically significant psychological distress between grandparents and the general population with a power of > 0.80 (alpha 0.05; two-sided) [44].

### Ethical approval and consent to participate

The study was approved by the Ethical Commission in Northwest and Central Switzerland (EKNZ 2020–01409, 23 September 2021). This study complies with the Swiss Federal Laws on data protection (235.1 Federal Act of June 19, 1992, on Data Protection) and the Swiss Human Research Act (810.30 Federal Act of September 30, 2011, on Research involving Human Beings).

Grandparents sign an informed consent form before receiving the questionnaire set (Study 1 and Study 3). Informed consent forms will be obtained on families enrolled in Study 1 signed by at least one adult in the family. Another informed consent will be obtained for interview participants stipulating consent for audio recording and storage (Study 2). Finally, for the study team to access medical records, parents sign informed consent if the child is < 14 years old, and provide written informed consent to their child signing an informed consent if the child is < 16 years. Children age 14 years and older sign their own informed consent. Electronic data are kept under the secured servers of the University of Lucerne, and any patient-related information is kept in a secured locker at all times. Any identifying information will be removed before data analysis to maintain anonymity.

## Discussion

Studies in the aging population are often challenging. For our research, we applied some best practices based on the literature to maximize enrollment and encourage participation. First, childhood cancer is a rare disease with an incidence of 14.1/100,000 children [45], making the identification of grandparents particularly difficult. Thus, indirect recruitment is the most practical approach. We initially identify the child with cancer, followed by tracing their parents, who subsequently refer grandparents to our study. However, it involves a series of informed consents (informed consent from the patient, parents, and grandparents) that may seem to the respondents as repetitive, yet a requirement by ethics commission. Second, we use post as a way to communicate the enrollment process. While electronic means have been predominantly utilized for data collection in most studies, prior research has demonstrated that adopting a more personalized approach enhances participation among the aging population [46]. We implemented both electronic and paper/pencil data collection, and the overwhelming majority, thus far, prefer the paper/pencil over electronic questionnaire completion. Third, our study materials are also tailored for the aging population. Information sheet includes statements that appeal to empathy, availability of study psychologist for counseling, and contact details of study staff ready to assist in filling out questionnaires. All these components convey an atmosphere of expertise, trust, support, and professionalism, theoretically encouraging participation [47–49]. Finally, the interview is an essential component of the study. Interviews will enable us to triangulate our findings from surveys and discover new domains that were not yet studied in the literature. To date, we have observed a high interview participation rate. Our participants, so far, have been receptive to the interviews and are willing to provide their time to share their experiences.

We have foreseen critical challenges that are a potential source of bias and could compromise the study conduct, namely, the delicate/emotional nature of the topic, the older adults’ declining cognition, participation on indirect recruitment, and drop-outs in longitudinal studies. First, childhood cancer is a psychologically sensitive topic that may trigger negative emotions, further hindering participation. As such, we provided the participants access to a psychologist if needed. Also, older adults may have a limited cognitive or physical capacity for surveys [46, 50]. Some may have cognitive impairment due to aging. Others may be technologically incapable of answering an electronic survey or have difficulties in writing due to a medical condition. Some may have limited literacy skills, considering mandatory education may not have been enforced during their childhood. To overcome this issue, we provide study staff contact details should they require assistance in answering the questions. Nevertheless, this problem is inherent in this age group and can be challenging to account for. Another anticipated challenge was participation following certain circumstances not controlled by the study team because of the indirect recruitment. Some families may not be invited to participate because they deem emotionally unstable to join a study. Some parents may also opt out sending information to grandparents because of dysfunctional or distant relationships. This would be mitigated by exploring the emotions and relationships deeper through interviews (Study 2). Otherwise, this is a recognized limitation of our enrollment design, as the alternative strategy of inviting all elderly in Switzerland and identifying those with a grandchild’s cancer is logistically impossible to implement. Finally, dropouts are expected to be higher in this population [50]. As such, we employ some cohort retention strategies to keep them engaged and, more importantly, to check if they need any psychological help.

Our study will collect one of the most comprehensive datasets on this topic as of the time of writing [8]. Our results will provide high-quality evidence as we employ a mixed-methods approach using various methodologies and sources. We will be able to provide robust effect estimates (through quantitative Studies 1 and 3), and a deeper context of the data through the grandparents’ lived experience (through the qualitative Study 2). We also collect data from the child with cancer, siblings, and the parents taking the whole family in context, which is rarely done in the literature. Another critical feature of our study is the longitudinal approach that could explore the temporality of psychosocial changes. Physical, mental, and social health are dynamic outcomes that change over time. Also, longitudinal studies are important in deriving any causal associations by considering the baseline measure to its outcome. Finally, this population-based, nationwide study will enroll grandparents from all over the country with high projected sample size, enabling us to perform more complex models, which have not been attempted due to sample size limitation in the literature.

The psychosocial impact of a grandchild’s illness has been gaining importance due to the increasing aging population and the increasing involvement of older adults in family dynamics. Our study will give insight into the impact of childhood cancer on a neglected group of family members. We will learn about the well-being and psychosocial health of grandparents of childhood cancer patients and survivors. Furthermore, the study will provide important information about the costs associated with the care provided by grandparents. Our findings will provide insights about where, when, and how to implement specific services that will help support those who provide vital support to families with a child diagnosed with cancer.

### Supplementary Information

**Additional file 1.**

## Data Availability

Data will be made available to interested parties upon reasonable request from the corresponding author.

## Abbreviations

BFI-K: Big Five Inventory of personality
BSI-18: Brief Symptom Inventory short form (18 items)
CCFU: Childhood Cancer Follow-up study
CD-RISC: Connor-Davidson Resilience Scale
EQ-5D-5L: European Quality of Life questionnaire
FaBel: Familien-Belastungs-Fragebogen (Family relationships questionnaire)
HLS-EU-Q12: European Health Literacy Survey Questionnaire (12 items)
HRQoL: Health-related Quality of Life
IES-R: Impact of Event Scale revised questionnaire
MSPSS: Multidimensional Scale of Perceived Social Support
PSS: Perceived Stress Scale
PTG-I: Post Traumatic Growth Inventory questionnaire
SF36: Health-Related Quality of Life Short Form Survey
WAQ: Worry and Anxiety Questionnaire
