# Supplementary material for: Acute and long-term psychosocial consequences in grandparents when a grandchild is diagnosed with cancer – the GROKids Project: a population-based mixed-methods study protocol
Source: BMC Psychol. 2023 Sep 18;11:280. doi: 10.1186/s40359-023-01309-w (PMC10507913; doi:10.1186/s40359-023-01309-w)
Supplement: Supplementary file 1 — Additional file 1. [file 40359_2023_1309_MOESM1_ESM.docx]

**Appendix**

**Table S1.** Structure of interview guide for the qualitative study (study 2).

General information

- General socio-demographic information

Experiences

- How did you experience the diagnosis in your grandchild?
- How did you experience the time of treatment of your grandchild?
- How have you been affected by the cancer diagnosis?

Grandchild care, help and support

- Have you been involved in the care of your grandchild with cancer? If yes, how?
- Have you been involved in the care of your grandchildren (siblings) without cancer? If yes, how?
- Did you provide help and support to your children (parents of child with cancer)? What type of help and support did you provide?
- Did you experience support needs yourself? If yes, what support needs?

Health and well-being

- Have your health and well-being been affected by the cancer diagnosis? If yes, how?

Daily life and employment

- Has your daily life and employment / retirement been affected by the cancer diagnosis? If yes, how?

Relationships

- Have your relationships been affected by the cancer diagnosis? If yes, how?
  - With your grandchild with cancer
  - With your other grandchildren (siblings of child with cancer)
  - With your child (parent of child with cancer)
  - With your partner
  - With friends

Advice to other grandparents and positive outcomes
